# Case report of successful treatment of patient with ruptured celiac artery aneurysm

**DOI:** 10.3389/fsurg.2025.1602499

**Published:** 2025-07-10

**Authors:** I. A. Semenenko, V. A. Yumasheva, N. Alieva, N. V. Yasnopolskaya, V. M. Sysoev

**Affiliations:** ^1^ University Clinic of General, Reconstructive and Cardiovascular Surgery, I.M. Sechenov First Moscow State Medical University of the Ministry of Health of Russia (Sechenov University), Moscow, Russia; ^2^ University Clinic of General, Reconstructive and Cardiovascular Surgery, Moscow State Budgetary Healthcare Institution “Moscow City Hospital Named After S.S. Yudin, Moscow Healthcare Department”, Moscow, Russia

**Keywords:** aneurysm, celiac artery, rupture, endovascular, stent graft

## Abstract

Celiac artery aneurysms (САА) represent the fourth most common visceral artery aneurysm. Despite its rarity, CAA carries a definite risk of rupture and/or other serious complications, which can be fatal. The reported rupture risk varies in the scientific literature, but it appears to range from 10% to 20%. CAA is often diagnosed at a late stage, after it has ruptured. There is currently no consistent approach to managing patients with CAA or its rupture. The aim of this case report is to present a successful minimally invasive treatment of CAA rupture.

## Introduction

Celiac artery aneurysm (CAA) is a rare aneurysm, accounting for 0.1%–2% of all visceral artery aneurysms and representing the fourth most common type (1, 2). CAA is associated with a formidable complication such as rupture, which carries the risk of a fatal outcome. The reported rupture risk varies in the scientific literature, but it appears to range from 10% to 20% (2). Early diagnosis and treatment of the disease is crucial to prevent the development of life-threatening complications. However, to date, there is no uniform strategy for managing patients when CAA or its rupture is detected, which underscores the importance of this issue for both practitioners and researchers in the field of vascular surgery (1, 3, 4). The aim of this case report was to present a successful minimally invasive treatment of CAA rupture.

## Case description

A 44-year-old man was admitted to the department of purulent surgery of Moscow State Budgetary Healthcare Institution “Moscow City Hospital named after S.S. Yudin, Moscow Healthcare Department” with an abscess of the left foot and right forearm. The patient underwent surgical intervention, which included opening and draining the abscesses, as well as treating the wounds. The patient also received drug therapy, including empiric antibacterial therapy.

Additionally, the patient had a history of chronic glomerulonephritis (morphologically focal segmental glomerulosclerosis), nephrotic syndrome, and chronic kidney disease (CKD) C1A4 (glomerular filtration rate of 111 ml/min according to the CKD-EPI equation). The patient also had steroid-induced diabetes and a left medial ankle fracture with metal osteosynthesis involving a plate and screws. Trauma and surgery data were unavailable. According to the patient, there was no aggravation of family history, including hereditary diseases. Notably, the patient had no history of abdominal infection or trauma.

On day 6 of the hospitalization, the patient's clinical blood test showed a significant decrease in hemoglobin (Hb) to 82 g/L (Hb was 111 and 96 g/L on admission and on day 5 of the hospitalization, respectively) and hematocrit (Ht) to 24.8% (Ht was 30.2 and 28.4% on admission and on day 5 of the hospitalization, respectively). Upon examination, the patient appeared unstressed and complained of weakness but did not have abdominal pain or tenderness. The heart rate was 92 beats per min, and the blood pressure was 100/60 mmHg. Therefore, the shock index was 0.92, corresponding to a blood loss of 20% of the circulating blood volume. Based on the clinical and laboratory data, internal bleeding was suspected. To confirm the origin of the bleeding, we performed a computed tomography (CT) of the chest, abdomen, and small pelvis with intravenous contrast.

**Figure 1:**
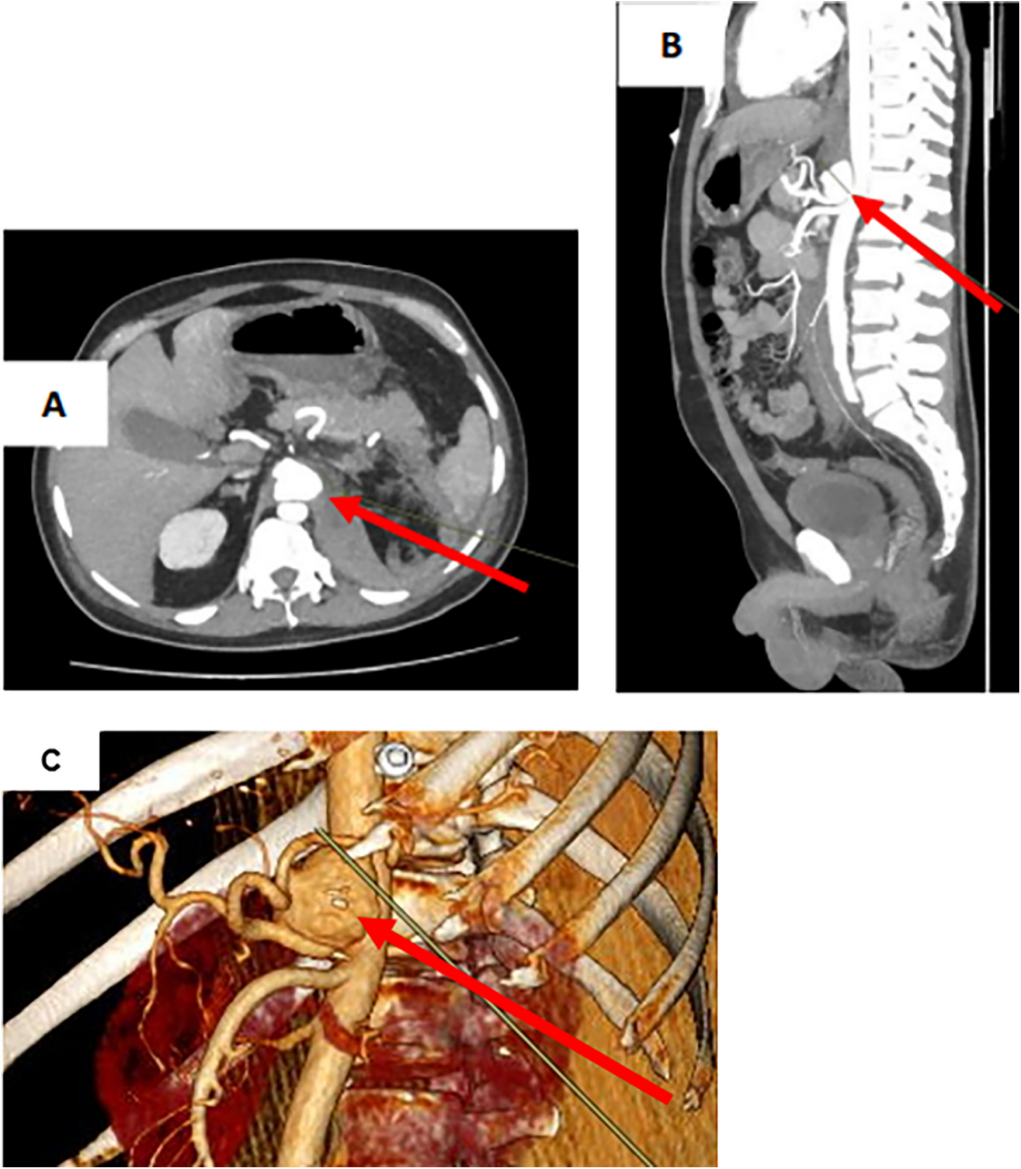
Ct scan of the abdomen with intravenous contrast in CAA rupture. **(A)** CAA in transverse projection. **(B)** CAA in sagittal projection. **(C)** CAA on 3D model. CAA (arrow).

CT angiography revealed a retroperitoneal hematoma and identified an irregularly shaped celiac artery aneurysm as its source. The aneurysm measured 33.5 mm × 24.5 mm × 38 mm (Figure 1). However, there was no evidence of contrast extravasation at the time of the study. The aneurysm was located 5 mm from the celiac artery orifice. A strong accumulation of hyperdense component was detected in the retroperitoneum on the left side, as well as para-aortically. These CT scans, in combination with clinical and laboratory data, were consistent with CAA rupture.

**Figure 2:**
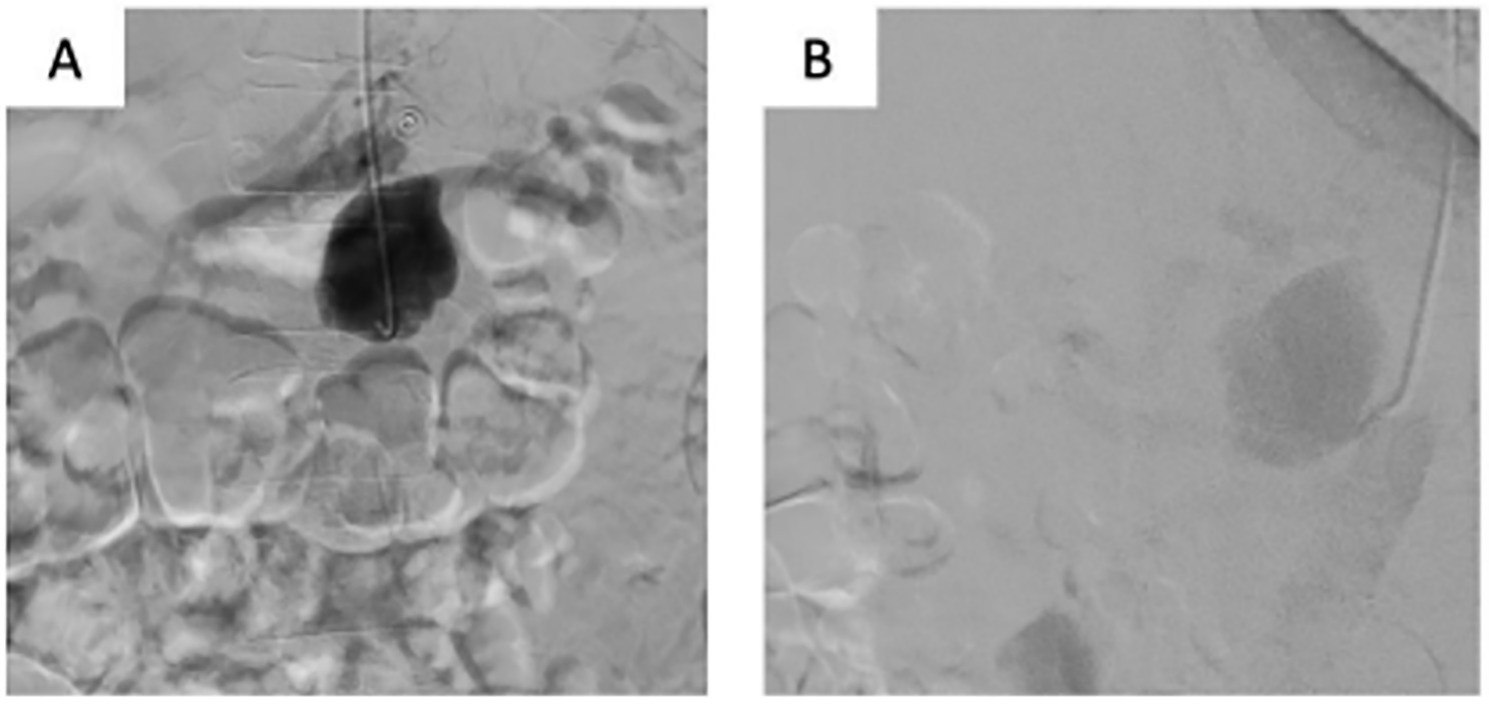
Endovascular aortography. **(A)** CAA. **(B)** Condition after stent graft implantation.

The patient underwent urgent CA angiography performed via a radial access. The CA angiography revealed a 45 mm × 30 mm saccular aneurysm in the proximal third of the artery (Figure 2). The BeGraft stent graft was then implanted using a 7 mm × 37 mm balloon catheter. Control angiography showed aneurysm occlusion and patency of the CA and all its branches.

**Figure 3:**
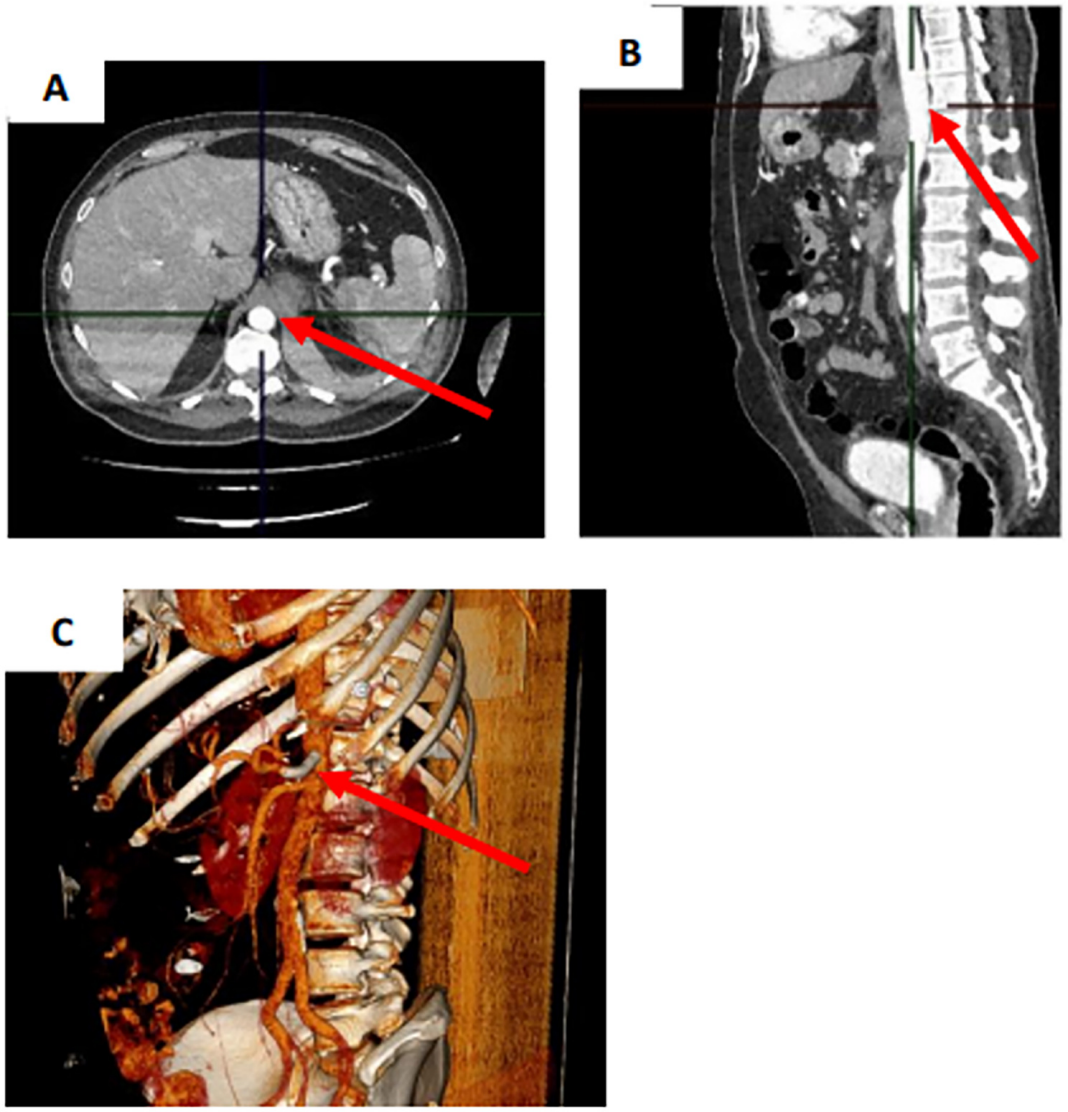
Control CT scan of the abdomen with intravenous contrast on the next day after stent-graft implantation. **(A)** Transverse projection. **(B)** Sagittal projection. **(C)** 3D model. CAA (arrow).

To evaluate the patient's condition in the postoperative period, the following examinations were performed: clinical and biochemical blood tests, a coagulogram, an abdominal aortic Doppler ultrasound, a Doppler ultrasound of the lower extremity arteries, and an ultrasound examination of the abdomen and retroperitoneum (Figure 3). According to the results of the laboratory and instrumental examinations, no impairments were observed in the patient. There were no adverse or unanticipated events. On day 16, the patient was discharged under outpatient supervision by the surgeon.

**Figure 4:**
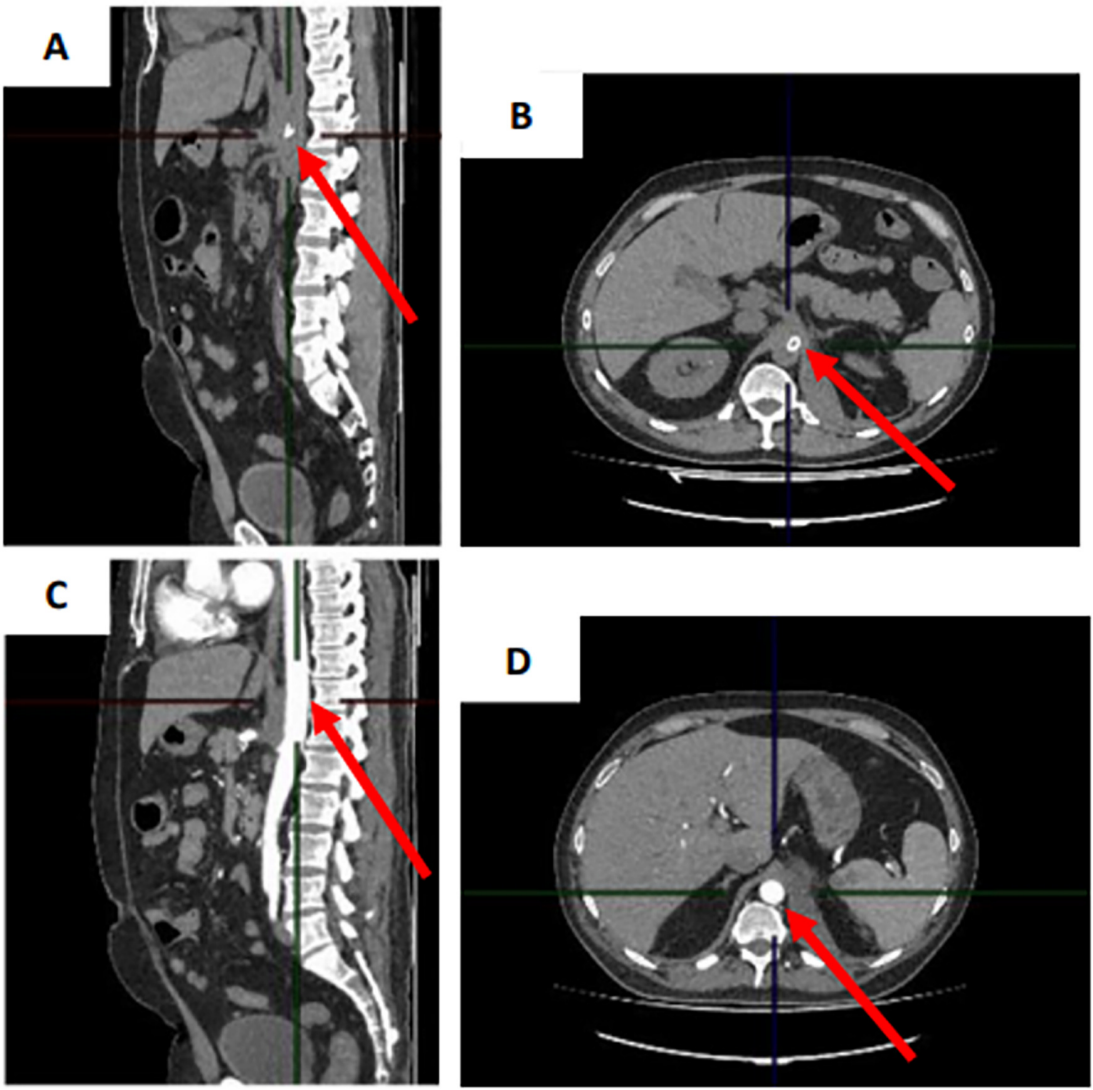
**(A,B)** CT scan of the abdomen with intravenous contrast in sagittal and transverse projection and **(C,D)** CT angiography scan of aorta and its branches with intravenous contrast in sagittal and transverse projection 3 weeks after stent graft implantation.

A control CT of the abdomen and a CT angiography of the aorta and its branches with intravenous contrast showed no contrast defects or leakage outside the vascular bed after 3 weeks, while regression of the retroperitoneal hematoma was observed (Figure 4).

## Discussion

The most common cause of CAA is atherosclerosis (1). However, other causes, such as infections, congenital diseases, trauma, and hereditary diseases associated with connective tissue weakness (e.g., Ehlers-Danlos syndrome), are also important (5).

CAA is classified into two types based on its anatomical location. Type I is an aneurysm of the CAA, and type II is an aneurysm of its branches. Each type is further subdivided into spindle-shaped and saccular aneurysms, according to their shape (6). In the case of our patient, type Ib CAA was present.

The high mortality rate among CAA patients is associated with late diagnosis and subsequent rupture, so early detection and treatment are crucial (7, 8). It is important to examine the vessels for an aneurysm, especially CAA, during a CT scan of the abdomen with intravenous contrast. Additionally, a differential diagnosis should be performed when examining patients, taking into account the presence of this rare pathology (1, 9).

The scientific literature describes three following treatments for CAA: conservative therapy, endovascular treatment with the possibility of using stent grafts or embolization, and open surgical treatment with ligation of the CA with or without revascularization (1, 3). However, there are no clear indications for choosing one treatment over another. However, the scientific literature discusses the following approaches: surgical treatment is recommended in cases of clinical symptoms, CAA > 2 cm, growth of CAA > 0.5 cm per year, and CAA in pregnant women or women of reproductive age, as these patients are most susceptible to aneurysm rupture (4, 10, 11). When a patient has indications for surgical treatment, the choice of treatment is strictly based on individual factors. It should be noted that open surgery is associated with a high mortality rate due to frequent complications. However, open surgery is a relevant treatment option when endovascular treatment is ineffective or inapplicable (9).

Fourteen clinical cases of CAA have been presented in the scientific literature published in the last 5 years, highlighting the importance of this issue (1, 3, 5, 7–9, 12–17). We have summarized the data and treatment strategies for these cases in Table 1. These articles propose different approaches to treating CAA patients. In 8 cases, open surgical interventions with aneurysm ligation and, in some cases, additional reconstructive procedures were performed. In one case, a palliative bleeding area package was performed due to an inability to stop the bleeding (1, 5, 8, 9, 12).

**Table 1:** Data from 14 clinical cases of CAA published in the scientific literature in the last 5 years.

Reference	Hospitalization	Hereditary disease	Anatomic anomaly of the visceral arteries	CAA size, mm	Treatment	Outcome
Vecchia et al. (1)	Urgent, with CAA rupture	—	—	32 × 24	Open surgery: the bleeding area was packed	Lethal (coagulopathy)
Oishi et al. (3)	Planned, with CAA	—	The celiacomesenteric trunk	39	Open reconstructive surgery: CAA was repaired with a saphenous vein graft	Improvement
	Planned, with CAA	—	The celiacomesenteric trunk	31	Open reconstructive surgery: CAA was repaired with a saphenous vein graft and an additional venous patch	Improvement
Ozawa et al. (5)	Planned, with CAA	—	—	84 × 54	Open surgery: aneurysmectomy and hepatosplenic anastomosis	Improvement
Bramucci et al. (7)	Urgent, with jaundice due to common bile duct compression by CAA	—	—	40 in diameter	Combined endovascular and endoscopic approach: stent graft and plastic stent implantation, respectively	Improvement
Znaniecki et al. (8)	Planned, with CAA	—	—	25 in diameter	Open surgery: resection and end-to-end anastomosis of the CA and common hepatic arteries	Improvement
Rajahram et al. (9)	Urgent, with CAA rupture	Neurofibromatosis type 1	—	—	Open surgery: CA was legated	Lethal (disseminated intravascular coagulation)
Bhandari et al. (12)	Urgent, with CAA rupture	Ehlers-Danlos syndrome	—		Open surgery: CA, splenic and hepatic arteries were ligated	Improvement
	Planned, with CAA	Ehlers-Danlos syndrome	—	17	Open surgery: CA was ligated	Recurrent pseudoaneurism (ligation was repeated)
Goto et al. (13)	Urgent, with CAA rupture	—	The celiacomesenteric trunk	26 × 28	Endovascular CA embolization	Improvement
Campbell et al. (14)	Urgent, with CAA rupture	—	—	10 × 5	Endovascular stent graft implantation	Improvement
Dwivedi et al. (15)	Planned, to detect a cause of abdomen pain	Behcet's disease	—	60	Endovascular stent graft implantation to the aorta and transcatheter coil aneurysm sac embolization	Improvement
Xiao et al. (16)	Planned, with CAA	—	—	105 × 97	Endovascular CA embolization	Improvement
Takata et al. (17)	Urgent, with CAA rupture	Neurofibromatosis type 1	—	26 in diameter	Combined endovascular stent graft implantation and laparotomic hemostasis	Improvement

An endovascular approach with embolization and/or stent-graft implantation was applied in 5 cases (3, 7, 13–16). One case involved a combined endovascular stent graft implantation and laparotomy hemostasis procedure (17). The type of surgical intervention was chosen based on CAA size, distance from the CA orifice, patient's comorbidities, and urgency (1, 3, 5, 7–9, 12–17). It should be noted that 2 cases had lethal outcomes due to late hospitalization and the development of irreversible and uncorrectable coagulation disorders (1, 9). One case presented with a recurrent pseudoaneurysm one year later, requiring repeated surgery (12).

We decided to perform an endovascular intervention with stent graft implantation because it is the fastest and least traumatic treatment. We also consider this method to be the best option for patients with comorbidities or emaciating conditions. Lastly, despite the saccular shape of the CAA, the patient had no signs of a current abdominal infection and no history of a previous one. The treatment was successful due to the timely diagnosis of CAA rupture. The endovascular intervention effectively repaired the CAA while preserving the patency of the CA, thereby minimizing risks to the patient.

Our study has the following limitations: there are no clear criteria for determining the optimal treatment for CAA. This makes it difficult to decide on a case-by-case basis. Long-term follow-up of patients is necessary to evaluate the stability of the results achieved and exclude late complications.

## Conclusion

Our report highlights endovascular intervention in a CAA patient to be an effective treatment. In addition, we consider stent-graft implantation to be the method of choice in comorbid and emaciated CAA patients.

## Data Availability

The data analyzed in this study is subject to the following licenses/restrictions: We present a case report. Requests to access these datasets should be directed to Valentina Yumasheva, valentina-jumasheva@rambler.ru.
